# Facilitators of high-quality teaching in medical school: findings from a nation-wide survey among clinical teachers

**DOI:** 10.1186/s12909-017-1000-6

**Published:** 2017-09-29

**Authors:** S. Schiekirka-Schwake, S. Anders, N. von Steinbüchel, J. C. Becker, T. Raupach

**Affiliations:** 1 Division of Medical Education Research and Curriculum Development, Study Deanery of Göttingen Medical School, Göttingen, Germany; 2 Department of Legal Medicine, University Medical Centre Hamburg-Eppendorf, Hamburg, Germany; 3 Institute of Medical Psychology and Medical Sociology, University Medical Centre Göttingen, Göttingen, Germany; 4 Department of Medical Education, Medical Faculty, University of Münster, Münster, Germany; 5 Department of Cardiology and Pneumology, University Medical Centre Göttingen, Göttingen, Germany

**Keywords:** Undergraduate medical education, Clinical teacher, Academic status, Survey, Questionnaire, Barriers, Facilitators, Didactic training, Faculty development, Evaluation

## Abstract

**Background:**

Clinical teachers in medical schools are faced with the challenging task of delivering high-quality patient care, producing high-impact research and contributing to undergraduate medical education all at the same time. Little is known on the gap between an ‘ideal’ environment supporting clinical teachers to provide high quality teaching for their students and the reality of clinical teaching during worktime in the clinical environment. Most quantitative research published so far was done in a wide range of medical educators and did not consider individual academic qualifications. In this study, we wanted to survey clinical teachers in particular and assess the potential impact of individual academic qualification on their perceptions.

**Methods:**

Based on qualitative data of focus group discussions, we developed a questionnaire which was piloted among 189 clinical teachers. The final web-based questionnaire was completed by clinical teachers at nine German medical schools.

**Results:**

A total of 833 clinical teachers (569 junior physicians, 264 assistant professors) participated in the online survey. According to participants, the most important indicator of high quality teaching was “sustained student learning outcome” followed by “stimulation of interest in the subject matter”. Lack of time was the main factor impeding effective teaching (78%). Among the factors facilitating high-quality teaching, protected preparation time during working hours (48%) and more recognition of high-quality teaching within medical schools (21%) were perceived as most helpful. Three out of four teachers (76%) were interested in faculty development programmes directed at teaching skills, but 60% stated they had no time to engage in such activities. With regard to evaluation, teachers preferred individual feedback (75%) over global ratings (21%). Differences between assistant professors and junior physicians were found in that the latter group perceived their teaching conditions as more difficult.

**Conclusions:**

Lack of time is a major barrier against planning and delivering good clinical teaching in medical schools. According to our findings, the situation at German medical schools is particularly challenging for junior physicians. Creating an institutional culture in which teaching is regarded as highly as patient care and research is a prerequisite for overcoming the barriers identified in this study.

**Electronic supplementary material:**

The online version of this article (10.1186/s12909-017-1000-6) contains supplementary material, which is available to authorized users.

## Background

Undergraduate medical education needs to meet high standards, and medical schools carry the responsibility to equip future physicians with knowledge, skills and attitudes needed to provide state-of-the-art care for their patients. A considerable amount of teaching in the clinical phase of medical education is delivered by physicians working in university hospitals. Not all of them may have chosen this work environment because they wanted to become clinical teachers, and in most medical schools the completion of a didactic training programme is not a prerequisite for being allowed to teach medical students. But even if they are motivated and adequately prepared to engage in teaching activities, clinical teachers are faced with different challenges as they are expected to produce high-impact research, contribute to medical education and deliver high-quality patient care, virtually all at the same time [1]. Few studies have identified a beneficial impact of teaching assignments on physicians: Hartley et al. [2] reported a positive effect on general practitioners’ attitudes following contact with students. In addition, teaching might reduce stress related to the clinical workload [3]. However, a much more common finding is that medical teachers struggle with their teaching role and face a lot of difficulties [4–10]: For instance, semi-structured interviews in 22 teachers in the United Kingdom revealed concerns about insufficient support and institutional recognition of teaching as well as the inability to influence decisions related to medical education [5]. In an Australian study, clinical teachers expressed worries about the quality of patient care as educational activities intervened with their clinical duties [4]. Furthermore, an international web survey including 860 participants revealed a lack of academic recognition and financial support as well as a specific need for didactic trainings as main challenges for medical educators (*n* = 860) [7]. Consistent with this finding, 147 German medical teachers named poor academic recognition (53.5%) as well as low institutional (31.5%) and financial (28.4%) support as important challenges in medical education [6].

Available studies on the subject are heterogeneous regarding their methodology and participants: Some authors used qualitative methods [4, 5, 8, 9], which provide useful information but usually rely on very small samples thus limiting generalisability. Most quantitative research published so far was done in a wide range of medical educators including basic scientists, psychologists, physiotherapists and nurses [7]. Given that the professional background (e.g. medical, nursing, psychology) and the work setting (e.g. university hospital, private sector) is likely to impact perceptions of the teaching environment, restricting survey samples to clinical teachers appears useful for addressing specific research questions. Physicians involved in undergraduate medical teaching are usually assigned to various teaching formats including bedside teaching, small-group discussions, case-based learning, problem-based learning and lectures. Thus, research on their perceptions of the teaching environment should not be confined to one specific format [8, 10].

Physicians working in German university medical centres can be divided into two groups: junior physicians (from graduation to board registration) and assistant professors (mostly consultants experienced in research). Junior physicians are primarily involved in patient care. As both teaching experience and research output are required to progress to the stage of an assistant professor, their workload is particularly high. In contrast, assistant professors hold positions with more responsibility and self-determination.

It is generally assumed that all physicians working in university hospitals strive not only to provide high-quality patient care and contribute to advance medical research but also to help their students become excellent physicians themselves. However, data supporting this notion are scarce. In fact, there is no uniform definition of ‘high-quality teaching’, and although a number of facilitators of good teaching have been described, it is unknown whether junior physicians and assistant professors share the same views on what supports and what deters them from delivering high-quality teaching.

The aim of this study was to elicit clinical teachers’ understanding of high-quality teaching characteristics and to identify facilitators and barriers of delivering high-quality teaching. In addition, we assessed teachers’ perceptions of evaluation and their motivation to participate in didactic trainings. Moreover, we wanted to investigate the impact of academic qualification on clinical teachers’ views.

## Methods

### Undergraduate medical education in Germany

There are two different models for undergraduate medical education in Germany: ‘Traditional’ curricula are made up of a two-year pre-clinical phase and a three-year clinical phase. In so-called ‘reformed’ curricula, pre-clinical and clinical teaching are integrated throughout the first 5 years. In both models, the sixth year comprises three elective periods lasting 4 months each [11]. Clinical teaching is an important feature of both models, thus curriculum type was not considered a major independent variable for this study.

### Development and piloting of the questionnaire

Due to the fact that no suitable questionnaire was available for our research question we developed a new instrument.

Physicians involved in undergraduate medical education at one German medical school (Göttingen) were invited to participate in focus group discussions addressing their definition of high-quality teaching, perceptions of the actual teaching environment, evaluation processes and issues related to clinical training. Teachers of all specialties were included and both junior physicians and assistant professors were invited to participate. During spring 2014, four focus group sessions including five to seven participants each were conducted. Two groups were made up of junior physicians (*n* = 15; nine male), and two groups included assistant professors (*n* = 11; all male). Sessions were moderated by one of the authors (SS). Results were categorised based on qualitative content analysis [12] using MaxQDA (VERBI GmbH, Marburg, Germany). Trigger questions served as an orientation for coding, and subthemes were identified in an iterative process. Themes and subthemes were subsequently included in mind maps.

Based on the themes that emerged from focus group discussions we developed a questionnaire containing scaled items. Following iterative discussions with experts in questionnaire development, a first draft of the questionnaire was used for cognitive debriefings with five clinical teachers (three female, two male; three junior physicians, two assistant professors) and adjusted according to their comments.

In January 2015, the web-based questionnaire was piloted in 183 clinical teachers (114 junior physicians (57 male), 69 assistant professors (49 male); overall response rate 26%) at Göttingen Medical School. Due to ceiling effects for some items addressing the importance of facilitators and barriers (i.e., strong agreement was found for a number of items), we added questions asking participants to rank order the items according to their perceived importance.

The final questionnaire comprised six sections: (1) demographics (seven items), (2) characteristics of high-quality teaching (17 items), (3) barriers against delivering high-quality teaching (eight items), (4) factors facilitating high-quality teaching (nine items), (5) didactic training experience (16 items), (6) utility of evaluation results for the improvement of teaching quality (five items). In addition to the 34 scaled items (5-point likert scale with one indicating the most negative and five indicating the most positive response), there were 26 categorical items and two open questions. A translated version of the questionnaire is provided in the Additional file 1.

Between March 2015 and February 2016, study deaneries of medicals schools were contacted by one author (SS) and invited to take part in the survey. Finally, nine medical schools (situated in eight of the 16 German federal states) agreed to participate (between September 2015 and July 2016). The invitation including the introduction to the project and the link to the web-based questionnaire (EvaSys, Electric Paper, Lüneburg, Germany) were sent to eligible clinical teachers by the respective study deanery. All participating study deaneries used comprehensive mailing lists including contact data of a wide range of faculty members. Unfortunately, the exact number of clinical teachers actually working in university hospitals could not be derived from this list, thus thwarting the calculation of a response rate.

### Data analysis

Descriptive analysis of survey data was performed by computing percentages and calculating mean values (M) ± standard deviations (SD), as appropriate. Independent t-tests, χ^2^ tests and effect size measures (Cohen’s d) were used to compare data obtained from junior physicians to survey responses provided by assistant professors. Significance levels were set to 5%. Owing to the explorative nature of all analyses, Bonferroni corrections were not used.

### Ethics approval

The Institutional Review Board at Göttingen University Medical Centre (application number 20/4/14) waived ethics approval as the study protocol was not deemed to represent bio-medical or epidemiological research. We made every effort to comply with data protection rules. Study participation was voluntary and all data were collected anonymously.

## Results

Six of the participating medical schools taught in ‘traditional’ and three in ‘reformed’ curricula. All of them offered didactic training programmes for their faculty and determined that participation in didactic trainings is a prerequisite for promotion to a position equivalent to a lecturer (e.g. assistant professor).

A total of 1035 subjects completed the questionnaire. Of these, 106 were excluded because they indicated not to be involved in patient care. Another 96 were excluded because they did not provide information on their academic qualification. Thus, a total of 833 questionnaires were analysed (569 junior physicians (313 male); 264 assistant professors (187 male)). Table 1 presents participant characteristics by academic status (junior physicians vs. assistant professors).

**Table 1:** Participant characteristics (grouped by academic status). Discrepancies in numbers result from missing values

		Junior physicians (*n* = 569)	Assistant professors (*n* = 264)
Sex	Female	245	71
	Male	313	187
Specialty Board Certification	Yes	302	247
	No	264	17
Age	≤35 years	272	10
	36–45 years	186	87
	>45 years	97	160
Teaching Experience	≤5 years	325	10
	>5 years	239	253
Specialty	Anaesthesiology	79	10
	General Medicine & Paediatrics	48	27
	Internal Medicine	104	36
	Neurology & Psychiatry	67	29
	Surgery, Orthopaedics & Urology	87	46
	Other (includes Gynaecology, Ophthalmology, Pharmacology, Toxicology, Pathology, Dermatology etc.)	89	50

**Fig. 1:**
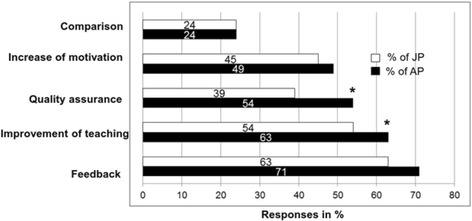
Utility of evaluation results for physicians (displayed by academic status). Columns represent percentages of survey participants who ticked the respective box on the survey. *p < 0.05 for the comparison of percentages (junior physicians, JP vs. assistant professors, AP) in a χ^2^ test

All other results are reported below, according to the thematic order of subjects in the questionnaire:

1. **Characteristics of high-quality teaching:** Ratings for the 14 scaled items on characteristics of high-quality teaching were heavily skewed towards the ‘very important’ anchor of the rating scale (e.g., high and sustained student learning outcome, good learning climate, students as well as teachers enjoy teaching sessions; see Table 2). While mean ratings for most items were >4.0 in both groups, the acknowledgement of individual differences between students was deemed less important by both junior physicians (3.41 ± 0.91) and assistant professors (3.44 ± 0.98). Significant differences in ratings between the two groups were found for “Teachers enjoy teaching/learning activities” (t(830) = 3.94, *p* < 0.001) and “Student learning outcome is high” (t(829) = 2.94, *p < 0.001*) in that assistant professors assigned greater importance to both aspects compared to junior physicians.
  When asked to indicate their individual most important characteristic of high-quality clinical teaching, 38% of the teachers chose “Student learning outcome is sustainable” followed by “Teacher motivates students and increases their enthusiasm for the subject matter” (16%) and “Students enjoy teaching/learning activities.” (12%). No significant differences between the two groups were found.
2. **Barriers against delivering high-quality teaching:** According to teacher ratings, the most important barrier against delivering high-quality teaching was a lack of protected preparation time while a lack of opportunity to implement individual approaches to teaching was the least important barrier (see Table 3). Junior physicians felt more strongly than assistant professors that they lacked protected preparation time for teaching (4.19 ± 0.98 vs. 3.88 ± 1.12; t (450.58) = 3.88; *p < 0.001*) as well as didactic training (2.68 ± 1.10 vs. 2.03 ± 0.90; t (608.09) = 9.03, *p < 0.001*).
  Analysis of the rank-ordering item revealed that lack of protected preparation time was the main factor impeding high quality teaching for both groups (chosen by 81% of all participants).
3. **Factors facilitating high-quality teaching:** Among the factors facilitating high-quality teaching, protected preparation time and positive student feedback were rated as being more important than individual incentives or time off as a reward for good teaching performance (see Table 3). There was a significant difference in ratings between junior physicians and assistant professors in that the former group was even more interested in protected preparation time (4.38 ± 0.95 vs. 3.98 ± 1.18; t(414.24) = 4.76, *p* < 0.001). Compared to assistant professors, junior physicians more strongly agreed that recognition for good teaching would help them deliver high-quality teaching (3.75 ± 1.12 vs. 3.45 ± 1.28; t(450.33) = 2.95, *p <* 0.001) and were more interested in time off as a reward for high quality teaching (3.27 ± 1.32 vs. 2.75 ± 1.44; t(467.56) = 4.93, *p* < 0.001).
  When asked to rank-order the five facilitating factors suggested in the survey, 49% (assistant professor = 44%, junior physicians = 51%) of participants chose protected preparation time.
4. **Didactic training experience:** Of all 833 survey participants, 483 (58%) had ever attended didactic training sessions with a significant difference between assistant professors (80%) and junior physicians (48%; (χ^2^(1, *N* = 828) = 77.72; *p* < 0.001). Over 82% were aware of their institution’s training programme, and 78% expressed a general interest in such programmes (junior physicians 84%; assistant professors 60%; χ^2^(1, *N* = 813) = 68.59; *p* < 0.001). The main reason for not participating in didactic trainings was a lack of time, as indicated by 64% of physicians (junior physicians 67%; assistant professors 56%; χ^2^(1, *N* = 806) = 9.07; *p* < 0.001*)*; barriers mentioned less frequently were lack of support from supervisors (12%; junior physicians 14%, assistant professors 6%; χ^2^(1, *N* = 806) = 12.26; *p* < 0.001), the notion that such trainings were not helpful (10% overall, no significant difference between groups), high cost (8% overall; *p* = n.s.) and lack of awareness of the availability of trainings (7%; junior physicians 8%, assistant professors 2%; χ^2^(1, *N* = 806) = 10.59; *p* < 0.001). A majority of teachers in both groups (76% overall (*N* = 817); *p* = n.s.) supported the view that participation in didactic trainings should be a prerequisite for promotion to a position equivalent to a lecturer (e.g. assistant professor). Content to be covered in trainings was rated on 5-point scales (see Table 4). Presentation skills were rated as most important by assistant professors (4.05 ± 1.51) whereas junior physicians were most interested in specific characteristics of teaching formats (4.04 ± 1.18). Again, ratings were skewed towards the ‘very important’ anchor of the rating scale. The only significant difference between assistant professors and junior doctors was observed for the item “Overview of the medical education system in Germany” (3.00 ± 1.29 vs. 3.24 ± 1.24; t (822) = 2.49, *p* = 0.01).
5. **Utility of evaluation results:** Only around one third (32%) of all survey participants (junior physicians = 26%, assistant professors = 44%) indicated to receive their evaluation results on a regular basis, whereas 24% (junior physicians = 30%, assistant professors = 12%;) never receive their results. The differences in frequencies were significant (χ^2^(2, *N* = 822) = 39:54; *p* < 0.001). Three in five teachers (60% (junior physicians = 55%; assistant professors = 72%; χ^2^(1, *N* = 764) = 21.21; *p* < 0.001)) stated they had tried to improve their teaching based on evaluation results.
  Most participants (66%) mainly used evaluation data as a source of feedback and for the improvement of their teaching (57%). About half of them (47%) felt that evaluation results increased their motivation to teach, and just one quarter (24%) used the data for comparisons with fellow teachers. Differences between assistant professors and junior physicians are displayed in Fig. 1. Significant differences were found for “Quality assurance” (χ^2^(1, *N* = 828) = 15.97; *p* < 0.001) und “Improvement of teaching” (χ^2^(1, *N* = 828*)* = 4.98, *p* = 0.03).
  With regard to type of evaluation, 75% of survey participants preferred individual evaluations over course evaluations (61%) and learning outcome evaluations (42%). Only 20% favoured global ratings.

**Table 2:** Characteristics of high-quality teaching. Items were rated on a 5-point scale (1 = very unimportant; 5 = very important)

Item	Mean ± Standard Deviation		Effect size (Cohen’s d)
	Junior physicians	Assistant professors	
Students enjoy teaching/learning activities	4.48 ± 0.68	4.53 ± 0.70	
Teachers enjoy teaching/learning activities	4.20 ± 0.78^a^	4.42 ± 0.69	−0.29
The learning climate is good	4.60 ± 0.65	4.60 ± 0.66	
Sessions have a clear structure	4.60 ± 0.69	4.69 ± 0.66	
Content is presented in a balanced manner	4.24 ± 0.79	4.33 ± 0.82	
Both knowledge, skills and attitudes are being taught	4.42 ± 0.80	4.39 ± 0.79	
Teachers agree in advance on the content to be taught	4.23 ± 0.79	4.16 ± 0.84	
Teaching format is aligned to learning objectives	4.26 ± 0.78	4.31 ± 0.77	
Teaching is pitched to the student level	4.10 ± 0.83	4.21 ± 0.79	
Teachers acknowledge individual differences between students	3.41 ± 0.91	3.44 ± 0.98	
Teacher motivates students and increases their enthusiasm for the subject matter	4.45 ± 0.74	4.51 ± 0.72	
Teachers have received didactic training	3.97 ± 0.93	4.05 ± 0.91	
Student learning outcome is high	4.14 ± 0.79^a^	4.31 ± 0.74	−0.23
Student learning outcome is sustainable	4.62 ± 0.69	4.63 ± 0.69	

^a^ *p* < 0.05 for comparisons between junior physicians and assistant professors (independent t test). Effect size (Cohen’s d) reported when t test was significant

**Table 3:** Barriers and facilitating factors for the delivery of high quality teaching. Teachers were asked to what extent these factors prevented them from/assisted them in delivering high-quality teaching. Items were rated on a 5-point scale (1 = not at all; 5 = very much)

Item	Mean ± Standard Deviation		Effect size (Cohen’s d)
	Junior physicians	Assistant professors	
Part I: Barriers			
Lack of preparation time	4.19 ± 0.98^a^	3.88 ± 1.12	0.32
Insufficient coordination between/among teachers	3.55 ± 1.01	3.49 ± 1.11	
Lack of opportunity to implement individual teaching concepts	2.90 ± 1.05	2.89 ± 1.25	
Lack of didactic training	2.68 ± 1.10^a^	2.03 ± 0.90	0.65
Part II: Facilitating factors			
Recognition for good teaching performance	3.75 ± 1.12^a^	3.45 ± 1.28	0.22
Individual incentives	3.25 ± 1.19	3.12 ± 1.30	
Positive student feedback	4.10 ± 0.85	4.10 ± 0.98	
Time off as a reward for high quality teaching	3.27 ± 1.32^a^	2.75 ± 1.44	0.40
Protected preparation time during working hours	4.38 ± 0.95^a^	3.98 ± 1.18	0.38

^a^ *p* < 0.05 for comparisons between junior physicians and assistant professors (independent t test). Effect size (Cohen’s d) reported when t test was significant

**Table 4:** Perceived utility of topics in didactic trainings. Items were rated on a 5-point scale (1 = very unimportant; 5 = very important)

Item	Mean ± Standard Deviation		Effect size (Cohen’s d)
	Junior physicians	Assistant professors	
Overview of medical education in Germany	3.00 ± 1.29^a^	3.24 ± 1.24	−0.19
Specific characteristics of the home institution	3.74 ± 1.22	3.83 ± 1.16	
Teaching session planning	3.78 ± 1.26	3.73 ± 1.20	
Specific characteristics of teaching formats	4.04 ± 1.18	3.92 ± 1.19	
Meeting the needs of a diverse student population	3.82 ± 1.39	3.71 ± 1.30	
Educational psychology	3.80 ± 1.47	3.89 ± 1.42	
Medical education research	3.39 ± 1.46	3.52 ± 1.48	
Presentation skills	3.96 ± 0.67	4.05 ± 1.51	
Designing practical examinations	3.88 ± 1.28	3.76 ± 1.38	
Designing oral examinations	3.97 ± 1.32	3.97 ± 1.28	
Designing written examinations	3.83 ± 1.30	3.83 ± 1.30	

^a^ *p* < 0.05 for comparisons between junior physicians and assistant professors (independent t test). Effect size (Cohen’s d) reported when t test was significant

## Discussion

Clinical teachers participating in this study had a broad understanding of high-quality teaching. Findings from local focus group discussions and pilot testing of the questionnaire at Göttingen medical school were confirmed in the nation-wide survey. Overall, student learning outcome was deemed to be an important indicator of high-quality teaching. The most salient barrier against delivering good teaching was a lack of protected preparation time. Positive student feedback served as a strong motivating factor while individual incentives appeared to play but a minor role. Lack of time also prevented physician teachers from attending didactic trainings although such trainings were considered very important by a majority. Individual evaluation was preferred and received results were used to improve teaching by most survey participants. Differences between assistant professors and junior physicians were found in that the latter group perceived higher barriers in terms of lack of time, lack of didactic trainings, and lack of support provided by supervisors.

Poor teaching conditions due to competing duties for physician teachers appear to be an international [4–8] problem that has been identified quite some time ago [1, 5]. It is alarming that despite teaching being an integral part of clinical teachers’ workload in university hospitals, there is insufficient institutional support in terms of allocated time for teaching itself, preparation of teaching activities or improving teaching by participation in didactic trainings in many institutions. Moreover, the present data indicate that at least at those German medical schools included in this survey, there is room for improvement with regard to academic recognition of high-quality teaching and providing individual evaluation results to teachers. The comparisons between junior physicians and assistant professors suggest that more support is particularly important during the first years following graduation. Presumably, assistant professors have more possibilities to cope with these conditions due to their experiences (made in undergraduate education as well as made as supervisors in continuing medical education) and a higher grade of self-determination.

Solutions for these deficiencies are long overdue. While care for patients must be the top priority for physicians, universities must also ensure that teaching (i.e. training of future physicians who must be equipped with skills to care for patients themselves) can be adequately delivered. The authors feel that teaching should be supported and valued as equally important as patient care and research. This means there can be no trade-off between either of these goals, but all of them should count towards performance measures of individual clinical teachers.

Protected time for teaching activities (including preparation) is needed. However, in some institutions more staff may be required to meet this goal. Given the economic pressure faced by many university hospitals, the creation of additional jobs with a specific focus on teaching seems unlikely but desirable (e.g. creating roles of “clinical educators” whose scope of duties comprise not only teaching but also supporting and supervising junior physicians in their teaching activities). Another possible, more economic strategy could be to involve medical students who are interested in engaging in (peer-)teaching activities.

Furthermore, the establishment of a teaching culture in which academic recognition for excellence in teaching is as high as for outstanding research and patient care may create a stronger motivation for clinical teachers to strive for excellence in teaching. Annual teaching prizes and financial incentives for departments supporting clinical teachers’ participation in dedicated training programmes are just a few examples of how perceived value of teaching may be increased.

Additional ways to manage this change in institutional culture need to be identified [13].

### Strengths and limitations

In contrast to previous studies on this topic, we focussed on one specific group of teachers. While this impairs the generalisability to other healthcare professionals involved in medical education, our results help to better understand why physicians sometimes fail to excel in teaching and what needs to be done in order to improve the situation. A particular strength of our study was the sample diversity and size. We included clinical teachers of different medical schools located in eight different German federal states offering different curricula. Moreover, we distinguished between junior physicians and assistant professors.

Interpretation of our results is limited in that only subjective data were assessed and the above-mentioned results and differences between the two groups are based on perceptions only. Furthermore, we could not calculate a definitive response rate, because most participating medical schools were unable to provide exact lists of physicians involved in undergraduate medical education. Due to this fact, we were unable to perform a power analysis; as mentioned above, all statistical comparisons were exploratory in nature. Moreover, because of self-selection of the participating medical schools the sample might not be representative of all clinical teachers at German medical schools.

## Conclusion

Physicians involved in undergraduate medical education face a number of serious barriers against delivering high-quality teaching, especially at the beginning of their career. Creating an institutional culture valuing teaching as no less important than patient care and research is a prerequisite for overcoming the barriers identified in this study.

## Additional file

Additional file 1: Translated version of the Questionnaire. (PDF 133 kb)

## Abbreviations

AP: Assistant professors
JP: Junior physicians
M: Mean value
N: Sample size
SD: Standard deviation
SS: Sarah Schiekirka-Schwake
