# Supplementary material for: Facilitators of high-quality teaching in medical school: findings from a nation-wide survey among clinical teachers
Source: BMC Med Educ. 2017 Sep 29;17:178. doi: 10.1186/s12909-017-1000-6 (PMC5622577; doi:10.1186/s12909-017-1000-6)
Supplement: Additional file 1: — Translated version of the Questionnaire. (PDF 133 kb) [file 12909_2017_1000_MOESM1_ESM.pdf]

Mark ☐☒☐☐☐
Correction ☐■☐☒☐

Introduction

Dear teacher,

Thank you very much for taking 10 minutes of your time to answer the following questions. With your participation in the survey you will help us to better understand your situation as a clinical teacher in undergraduate medical education.

Section 1: Personal Data

Are you working as a clinical teacher in undergraduate medical education?	☐ Yes	☐ No	
Your sex	☐ Female	☐ Male	
Your age	☐ Younger than 26 years	☐ 26-35 years	☐ 36-45 years
	☐ 46-55 years	☐ Older than 55 years	
Specialty Board Certification	☐ Yes	☐ No	
Assistant professor	☐ Yes	☐ No	
For how long have you been working as a clinincal teacher (in undergraduate medical education)?	☐ Less than 2 years	☐ 2-3 years	☐ 4-5 years
	☐ 6-7 years	☐ 8-9 years	☐ more than 9 years

In which of the following specialty are you practicing?

☐ General Medicine	☐ Anaesthesiology	☐ Occupational / Community Medicine
☐ Ophthalmology	☐ Surgery	☐ Dermatology / Venereology
☐ Gynaecology / Obstetrics	☐ Otorhinolaryngology	☐ Human Genetics
☐ Hygiene / Microbiology / Virology	☐ Internal Medicine	☐ Paediatrics
☐ Clinical Chemistry / Laboratory Diagnostics	☐ Neurology	☐ Orthopaedics
☐ Pathology	☐ Pharmacology / Toxicology	☐ Psychiatry and Psychotherapy
☐ Psychosomatic Medicine and Psychotherapy	☐ Forensic Medicine	☐ Urology
☐ Other		

Section 2

The following section addresses your ideal conception of high-quality teaching.

Please rate on the five-point scale provided („very unimportant“ to „very important“) how the following aspects characterize high-quality teaching for you.

	very unimportant	rather unimportant	neutral	rather important	very important
**(1)** Students enjoy teaching/learning activities	☐	☐	☐	☐	☐
**(2)** Teachers enjoy teaching/learning activities	☐	☐	☐	☐	☐
**(3)** The learning climate is good	☐	☐	☐	☐	☐
**(4)** Sessions have a clear structure	☐	☐	☐	☐	☐
**(5)** Content is presented in a balanced manner	☐	☐	☐	☐	☐
**(6)** Knowledge, skills and attitudes are being taught	☐	☐	☐	☐	☐
**(7)** Teachers agree in advance on the content to be taught	☐	☐	☐	☐	☐
**(8)** Teaching format (seminar, lecture, bedside teaching etc.) is aligned to learning objectives	☐	☐	☐	☐	☐
**(9)** Teaching is pitched to the student level	☐	☐	☐	☐	☐
**(10)** Teachers acknowledge individual differences between students	☐	☐	☐	☐	☐
**(11)** Teacher motivates students and increases their enthusiasm for the subject matter	☐	☐	☐	☐	☐
**(12)**Teachers have received didactic training	☐	☐	☐	☐	☐
**(13)** Student learning outcome is high	☐	☐	☐	☐	☐
**(14)** Student learning outcome is sustainable	☐	☐	☐	☐	☐

Please indicate your top three priorities (with regard to high-quality teaching) from this list by ticking the appropriate boxes (numbers refer to the parenthetic numbers in the above list).

The highest priority has aspect number:

☐ 1	☐ 2	☐ 3
☐ 4	☐ 5	☐ 6
☐ 7	☐ 8	☐ 9
☐ 10	☐ 11	☐ 12
☐ 13	☐ 14	

The second highest priority has aspect number:

☐ 1	☐ 2	☐ 3
☐ 4	☐ 5	☐ 6
☐ 7	☐ 8	☐ 9
☐ 10	☐ 11	☐ 12
☐ 13	☐ 14	

Section 2 [Continuation]

The third highest priority has aspect number:

☐ 1 ☐ 2 ☐ 3
☐ 4 ☐ 5 ☐ 6
☐ 7 ☐ 8 ☐ 9
☐ 10 ☐ 11 ☐ 12
☐ 13 ☐ 14

Section 3

This section addresses your personal teaching situation as is.

Please indicate on the five-point scale provided ("not at all" to "very much") to what extent the following factors prevent you from delivering high-quality teaching.

	not at all	slightly	moderately	much	very much
**(1)** Lack of protected preparation time during working hours	☐	☐	☐	☐	☐
**(2)** Insufficient coordination between/among teachers	☐	☐	☐	☐	☐
**(3)** Lack of opportunity to implement individual teaching concepts	☐	☐	☐	☐	☐
**(4)** Lack of didactic training	☐	☐	☐	☐	☐

Of these, please indicate the three most important barriers against the delivery of high-quality teaching by ticking the appropriate boxes (numbers refer to the parenthetic numbers in the above list).

The most important barrier for me is the factor with number:	☐ 1<br>☐ 4	☐ 2	☐ 3
The second most important barrier for me is the factor with number:	☐ 1<br>☐ 4	☐ 2	☐ 3
The third most important barrier for me is the factor with number:	☐ 1<br>☐ 4	☐ 2	☐ 3

If you feel there are additional barriers that have not been included in our list please enter your comments in this box:

There are two more questions on your personal teaching situation:

Please indicate on the five-point scale provided ("not at all" to "very much")to what extent the following factors would facilitate the delivery of high-quality teaching.

Section 3 [Continuation]

	not at all	slightly	moderately	much	very much
**(1)** Recognition for good teaching performance (e.g. by the faculty, head of department or direct supervisors)	☐	☐	☐	☐	☐
**(2)** Individual incentives	☐	☐	☐	☐	☐
**(3)** Positive student feedback	☐	☐	☐	☐	☐
**(4)** Time off as a reward for high quality teaching	☐	☐	☐	☐	☐
**(5)** Protected preparation time during working hours	☐	☐	☐	☐	☐

Of these, please indicate the three most important factors that would facilitate your delivery of high-quality teaching by ticking the appropriate boxes (numbers refer to the parenthetic numbers in the above list).

The most important facilitator for me is the factor with number: ☐ 1 ☐ 2 ☐ 3 ☐ 4 ☐ 5

The second most important facilitator for me is the factor with number: ☐ 1 ☐ 2 ☐ 3 ☐ 4 ☐ 5

The third most important facilitator for me is the factor with number: ☐ 1 ☐ 2 ☐ 3 ☐ 4 ☐ 5

If you feel there are additional facilitating factors that have not been included in our list please enter your comments in this box:

Section 4

Have you ever attended didactic training sessions? ☐ Yes ☐ No

Are you aware of any didactic training programmes at your medical school? ☐ Yes ☐ No

If so, which of the following factors have prevented you from attending (multiple selection is possible)?

☐ At my medical school, no programmes are offered
☐ Programmes are offered, but I think that they are not helpful
☐ My supervisor does not support my participation
☐ I do not have time to attend
☐ The offered programmes are too expensive
☐ Other

Should participation in didactic trainings be a prerequisite for promotion to a position equivalent to a lecturer (e.g. assistant professor)? ☐ Yes ☐ No

Section 4 [Continuation]

Please indicate on the five-point scale provided ("very unimportant" to "very important") your perceived importance of various contents of teacher training programmes.

	very unimportant	rather unimportant	neutral	rather important	very important	I do not know
Overview of medical education in Germany	☐	☐	☐	☐	☐	☐
Specific characteristics of the home institution	☐	☐	☐	☐	☐	☐
Teaching session planning	☐	☐	☐	☐	☐	☐
Specific characteristics of teaching formats (lecture, seminar, bedside teaching, problem based learning etc.)	☐	☐	☐	☐	☐	☐
Meeting the needs of a diverse student population	☐	☐	☐	☐	☐	☐
Educational psychology	☐	☐	☐	☐	☐	☐
Medical education research	☐	☐	☐	☐	☐	☐
Presentation skills	☐	☐	☐	☐	☐	☐
Designing practical examinations (e.g. OSCE)	☐	☐	☐	☐	☐	☐
Designing oral examinations (e.g. Mini-Cex)	☐	☐	☐	☐	☐	☐
Designing written examinations (e. g. multiple-choice-questions, true/ false-items, key-feature-questions)	☐	☐	☐	☐	☐	☐

Section 5

Do you regularly receive the evaluation results of your teaching sessions? ☐ Yes, always ☐ Yes, sometimes ☐ No, never

If you selected one of the two positive options for the previous question, please indicate the utility evaluation results have for you (multiple selection is possible)?

☐ Feedback ☐ Improvement of my own teaching ☐ Quality assurance

☐ Comparison with fellow teachers ☐ Increase of my own motivation ☐ No benefits at all

Have you ever improved your teaching based on your evaluation results? ☐ Yes ☐ nein

Section 5 [Continuation]

In your opinion, which type of evaluation is most useful (multiple selection is possible)? Data collection tools may comprise various formats, e.g. discussions, scaled questionnaire items, free-text comments)

- ☐ Individual evaluation (the individual teacher is evaluated by specific criteria)
- ☐ Course evaluation (the teaching session is evaluated by specific criteria)
- ☐ Evaluation of learning outcome / learning outcome in percentages (the students learning outcame is evaluated / calculated)
- ☐ Global ratings (general evaluation of teaching sessions, teachers etc. by one rating)

Which evaluation format do you prefer for the assesment of your teaching perfomance (multiple selection is possible)?

- ☐ Scaled items
- ☐ Free-text comments
- ☐ Feedback by a coach / trainer
- ☐ Group feedback conversations with students

Thank you very much for taking the time to complete the questionnaire.
